# Communication barriers faced by pharmacists when managing patients with hypertension in a primary care team: a qualitative study

**DOI:** 10.1186/s12875-024-02349-w

**Published:** 2024-03-27

**Authors:** Reuben Tan, Ariffin Kawaja, Swee Phaik Ooi, Chirk Jenn Ng

**Affiliations:** 1 Yong Loo Lin School of Medicine, National University of Singapore, Singapore, Singapore; 2 SingHealth Polyclinics, SingHealth, Singapore, Singapore; 3 Duke-NUS Medical School, Singapore, Singapore

**Keywords:** Barriers, Hypertension, Communication, Pharmacists, Multi-disciplinary team, Primary care

## Abstract

**Background:**

As primary care pharmacists take on an increasingly important and collaborative role in managing patients with chronic diseases, communication barriers with patients and healthcare colleagues have emerged. This study aimed to explore the communication barriers faced by pharmacists when managing patients with hypertension in a primary care team.

**Methods:**

Twelve pharmacists working in five government primary care clinics were interviewed by a researcher using a topic guide. The interviews were audio-recorded, transcribed verbatim and subjected to thematic analysis.

**Results:**

Pharmacists’ management of patients with hypertension was found to be affected by communication challenges at three different levels: between pharmacists and patients, pharmacists and physicians, and physicians and patients. Barriers to communication between pharmacists and patients include language barrier, physical disabilities, medication brand changes, and specific challenges faced during video consultations. Barriers to communication between pharmacists and physicians include lack of access to patient information across institutions on the electronic medical records (EMR), inadequate and inappropriate documentation by physicians, and disruptive and ineffective phone calls by pharmacists to physicians. Barriers to communication between physicians and patients had a spillover effect on pharmacists; these barriers included language barrier, patients not discussing medication nonadherence with physicians, and conflicting advice given by physicians and pharmacists.

**Conclusions:**

The communication barriers pharmacists faced when managing patients with hypertension involved multiple stakeholders. Many of the challenges resulted in patients having difficulty understanding and adhering to their management plan. Effective interventions to foster stronger interprofessional relationships and create a conducive platform of communication should be developed to address these communication barriers.

## Background

Hypertension is on the rise in Singapore and across the world [1, 2], and primary care pharmacists in Singapore play an important role in the management of patients with hypertension. The latest National Population Health Survey conducted in 2019/2020 found that 1 in 3 (35.5%) Singaporean adults (aged 18–74) has hypertension, of whom nearly half of them have never been diagnosed, and among those who have been diagnosed and are on treatment, 2 out of 3 are poorly controlled [2]. Despite the availability of effective blood pressure-lowering medications and the evidence of lifestyle modifications in reducing blood pressure, implementation of clinical evidence in real-world clinical practice remains challenging [3, 4]. This poses a significant burden of disease and care, as hypertension, if uncontrolled, can result in complications such as coronary artery disease, stroke, and chronic kidney disease [5]. This will affect the quality of life of patients and their caregivers and impose a significant healthcare cost and burden on the health system [6].

Currently, most patients with hypertension in Singapore (84%) are managed in the primary care setting, with the remaining patients being managed at specialist outpatient clinics in hospitals. Most patients (50.5%) seek care at government primary-care clinics (or polyclinics), while 33.5% of patients seek care from over 2000 private general practitioners [2]. Singapore has 23 polyclinics, which are healthcare centres that provide subsidised primary care, including primary medical treatment, preventive healthcare, and health education [7]. Besides primary care physician consultations, polyclinics offer other services including in-house nursing care, pharmacies, medical social services, dietitian, physiotherapy, podiatry, basic clinical laboratory, diagnostic radiology, and dental services. Pharmacists in polyclinics take on many roles, including reviewing prescriptions, dispensing medications, providing professional advice to physicians and other healthcare professionals on matters relating to drug use and disease management, as well as providing medication counselling to patients. Beyond Singapore, the practice of pharmacists is evolving as more pharmacists become incorporated into primary care teams, with a transition from dispensing medications to playing a more collaborative and patient-centred role [8]. Furthermore, pharmacist interventions have been shown to improve blood pressure control [9].

The role that primary care pharmacists play often requires them to interact not only with patients but also with other healthcare professionals [10]. Studies have identified several challenges faced by pharmacists when interacting with their healthcare colleagues. One commonly faced barrier was “medical dominance”, as defined by the Health Sociology Review as the occurrence of physicians “exerting sovereign power over other professions such as nursing” [11], thus undermining and restricting the roles of pharmacists [12]. Lack of clarity regarding a pharmacist’s role and responsibilities by other healthcare professionals also led to underutilisation of their services [13]. Studies have also shown that some physicians show a lack of respect towards pharmacists, resulting in pharmacists avoiding interacting with physicians [14, 15]. Often, even trying to reach physicians proved to be challenging for pharmacists [16].

The pharmacists in Singapore’s polyclinics work in dynamic multidisciplinary teams to deliver care for a large number of patients who come from diverse socioeconomic, cultural and language backgrounds. Pharmacists work in a setting where one pharmacist will interact with multiple physicians who each have different communication and documentation styles. Furthermore, less than half of Singapore’s population (48.3%) uses English as their most frequently spoken language, and many other languages are spoken in this multiracial country, including Mandarin, Chinese dialects, Malay, Tamil, and other languages [17]. However, very few studies have been conducted locally to explore the communication challenges faced by primary care pharmacists in managing patients with hypertension.

We conducted a qualitative study exploring challenges faced by pharmacists when managing patients with hypertension in a Singapore public primary healthcare setting. ‘Communication barriers’ emerged as the main overarching theme. Therefore, this paper aimed to explore the communication barriers faced by primary care pharmacists when managing patients with hypertension in Singapore. By identifying these barriers, interventions can be developed to fill the gaps and address the unmet needs. The findings will be relevant to pharmacists who work in a team and manage patients with diverse backgrounds and to decision makers who are planning for healthcare interventions to improve hypertension care.

## Methods

### Study design

This study utilised a qualitative methodology consisting of individual in-depth interviews (IDIs), as it allowed us to inquire and delve into the views and experiences of primary care pharmacists concerning the management of patients with hypertension as encountered in their local practices [18].

### Setting

The public healthcare system in Singapore is grouped into three clusters based on geographical location, with SingHealth Polyclinics managing patients in the eastern region of Singapore [19]. The study was conducted among primary care pharmacists involved in managing patients with hypertension across five SingHealth Polyclinics in Singapore. The five selected Polyclinics were distributed across various locations in the eastern region of Singapore and had different patient population profiles (age, socioeconomic status, education level and health literacy). Care was also taken to ensure that a spectrum of practice experience was represented.

In the polyclinics, most of the patients seen by pharmacists are above 64 years old, Mandarin speaking, and taking five medications on average. Polyclinic patients can consult pharmacists for a “medication review”. Patients are identified for medication review via two channels. Patients with medication-related issue(s) can be referred by a physician in the polyclinic, subject to the physician’s clinical discretion. Alternatively, patients who had not been referred by a physician for medication review, but are collecting their medications at the pharmacy counter, can be assessed by the dispensing pharmacist to have medication-related issue(s) and can receive the review service, subject to the patient’s consent. Each medication review session may take about 20 to 30 min, depending on the complexity of the case. The medication review process involves the evaluation of therapeutic efficacy of each drug, unmet therapeutic needs and the progress of the condition being treated. Other areas such as patient’s compliance, understanding of their conditions/drug treatments, correct administration techniques, storage conditions, etc., are assessed and reinforced where necessary. Actual and potential adverse effects, interactions (e.g. drug-drug, drug-food and drug-herb) are also highlighted. Medication review sessions are not disease-specific but tailored to each individual patient. For example, a patient with hypertension and diabetes mellitus will be reviewed for both conditions, and not just for hypertension. Should any medication-related issue be identified, the pharmacist will discuss a proposed action plan with the patient, and the prescriber if required. Where appropriate, the pharmacist will make a telephone call to the patient two weeks after the medication review session to evaluate the outcome of the action plan and arrange for the date of the next medication review session.

### Participants, recruitment, sampling

We identified pharmacists at SingHealth Polyclinics who were involved in the care of patients with hypertension for at least six months. We purposively sampled pharmacists of different levels of seniority with varying years of experience practising in primary care. In addition, a pattern of snowball sampling developed as the participants named other pharmacists whom they felt would provide additional insights. Sample size was determined by data saturation whereby interviews were stopped when no new ‘views’, ‘experiences’, or ‘challenges’ emerged from the interviews and data analysis.

### Data collection

The researchers of this study are RT, NCJ, AK and OSP. RT is a medical student; NCJ is a professor and clinician who specialises in family medicine; AK is a research fellow with a PhD degree in health communication; and OSP is a senior pharmacist working in a SingHealth Polyclinic.

Before commencing the IDIs, an interview topic guide was developed based on a literature review, clinical knowledge and research experience (Table 1). All interviews were carried out by NCJ, who had 20 years of experience conducting qualitative research. We avoided, whenever possible, selecting participants who were close acquaintances or colleagues of NCJ to minimise potential participant response bias. Prior to the interviews, the researchers gave the study information sheet and explained to the participants the aims of the study and the research method before informed consent was obtained from the participants. During the interviews, RT took detailed field notes that were used as discussion and comparison pointers with NCJ after the interviews, as well as for data analysis later. No repeat interviews were conducted. From March to October 2022, twelve 40–60 min IDIs were conducted. Data collection was stopped when data saturation had been reached. All IDIs were audio-recorded and transcribed verbatim, after which they were checked for accuracy and used as data for analysis.

**Table 1:** Study interview topic guide

1. Can you tell me about the patient profile at your polyclinic? a. Age group, ethnicity, education level, disease severity, health-seeking behaviour
2. How do patients with hypertension first present in your clinic?
3. Do you use any clinical practice guideline to guide your hypertension management? a. If yes, which guideline and why b. What do you think about the guideline? Useful or not? Why?
4. What is your approach to treating hypertension? a. Probe: Nonpharmacological – diet, exercise (be specific) b. Probe: Medications – which category, order of starting, mono- or dual therapy, how and when to step up
5. What are the challenges you face when managing patients with hypertension? a. Probe: Patient factor: Nonadherence to treatment/lifestyle modifications, communication barriers etc. b. Probe: Pharmacist factor: Knowledge and skills, keeping updated with latest evidence, Communication with other healthcare professionals etc. c. Disease factor: Diagnosis of different categories of hypertension (e.g. white-coat, masked, nocturnal, non-dipper, morning surge), etc. d. System factor: Use of electronic medical records for patients with hypertension, PTEC, interprofessional care delivery, etc.
6. Can you suggest ways to improve the current management of hypertension? a. Probe: Change in current hypertension care delivery? Training? Technology? b. Probe: Do you need any support to provide better care for your patients
7. Do you have anything else to share with me?

### Data analysis

After transcription and checking, the transcripts were imported into NVivo computer-assisted qualitative data analysis software for data management and thematic analysis. Inductive coding was then performed. Initially, two researchers each analysed one transcript independently. Codes (short phrase labels) were assigned to specific data sections that represented their significance (open coding). Subsequently, the researchers used their developed codes to code one more transcript each. The coding was then compared for inter-researcher consistency. Any differences were resolved by discussion until an agreement was reached on the list of codes. This list was then reviewed, and the codes were grouped together to form categories (axial coding). These categories were further reviewed in terms of relation, and underlying themes were created to reflect the meaning of the data. This coding framework was then used to code data from the remaining transcripts [20].

The remaining transcripts were distributed among three researchers (RT, NCJ, AK) and coded individually. New codes that emerged during analysis were added to the list upon consultation with the other researchers, while those that were not relevant were removed. The coding framework was continually relooked, and codes were rearranged into different or new categories as deemed appropriate through discussion among the researchers. In alignment with the comparative case approach, which used assorted forms of data [21], field notes taken during the interviews were also reviewed and included in the analysis process to help in comprehension and clarification of the data.

Data analysis was conducted from both clinical (RT and NCJ) and nonclinical (AK) perspectives. Although member checking was not conducted, our findings were presented to the fourth researcher (OSP) for review. OSP, who is a primary care pharmacist, checked and critiqued the analysis. Her feedback was subsequently used to revise the interpretation of the data. To improve the credibility of the analysis, the research team caried out regular reflection and discussion on potential biases that the researchers might harbour due to their backgrounds.

## Results

A total of 12 pharmacists participated in the study. Table 2 shows the participants’ demographic data.

**Table 2:** Demographic profile of participants

Characteristics	Number (*n* = 12)	%	Mean ± SD (Range)
Age			39.5 ± 9.5 years (26–61 years)
Sex			
Female	7	58.3	
Male	5	41.7	
Years of experience			14.1 ± 9.78 years (2–35 years)
Polyclinic			
C1	2	16.7	
C2	3	25	
C3	2	16.7	
C4	3	25	
C5	2	16.7	

The initial research question was to broadly explore the challenges and barriers faced by primary care pharmacists in the management of patients with hypertension. However, one recurrent theme emerged very strongly across all five polyclinics: communication.

**Fig. 1:**
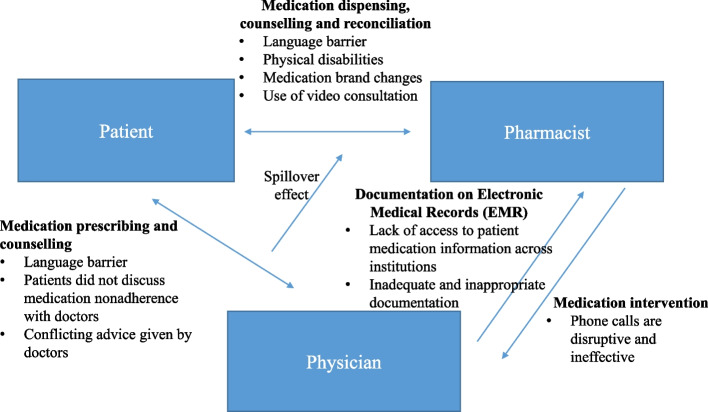
Communication barriers faced by primary care pharmacists in the management of patients with hypertension

With different stakeholders involved in the management of patients with hypertension, pharmacists were affected by communication challenges at three different levels: between pharmacists and patients, pharmacists and physicians, and physicians and patients. Figure 1 illustrates the barriers that surfaced at each interaction.

### Between pharmacists and patients

Pharmacists interacted with patients in the setting of medication review, dispensing, counselling, and reconciliation. Challenges emerged during these interactions that impacted the pharmacist’s management of patients with hypertension.

#### Language barrier

Language was a commonly raised factor. As a multiracial society, Singapore is home to many different ethnicities that speak varying languages; hence, language barriers emerged between pharmacy staff and patients, and this affected their ability to counsel patients appropriately. As put across by one pharmacist,

“Because many times they (patients) will tell us things like, ‘Oh, because when I got the medicine, I got it from a Malay speaking staff and I’m a Chinese and I don’t understand their English’”

- Pharmacist with 15 years’ experience, Polyclinic C4

One pharmacist also pointed out that pharmacy staff would turn to other staff in the polyclinic to help with translation. This highlighted the issue of accuracy of translation, as medical and drug-related counselling has to be as accurate as possible to prevent medication errors and patient misconceptions.

“For example, we only have one or two Tamil-speaking staff; if this one patient only speaks Tamil, we may actually approach cross-domain staff who are Tamil-speaking to help us with the translation. However, we have to be more careful, because sometimes when they help us to translate, we do not know whether they actually translated it correctly or not.”

- Pharmacist with 15 years’ experience, Polyclinic C2

#### Patients with physical disabilities

Many pharmacists mentioned that a number of patients with hypertension they encountered had disabilities that made communication more challenging. Deficits in patients’ memory, hearing, and vision caused patient counselling to become more time-consuming and at times ineffective.

“I would say it happens mainly to the very old elderly or maybe they might have Alzheimer’s or dementia or poor memory. That kind of patient, we will need more time. They tend to forget what the physician said, so they need more time to counsel.”

- Pharmacist with 2 years’ experience, Polyclinic C1

“However, there can be patients who wear hearing aids; they may not be able to hear us properly. So I tend to write (the instructions) for them, if there’s a need to.”

- Pharmacist with 2 years’ experience, Polyclinic C2

#### Medication brand changes

According to the pharmacists, medication brand changes were a common occurrence that made communication with patients even more arduous. Owing to polyclinic policy and partially due to supply chain disruptions during the COVID-19 pandemic, medications stocked in the pharmacy underwent brand changes regularly. When asked how frequently these medication brand changes occurred, one pharmacist replied,

“It can be every few months, every 3-4 months.”

- Pharmacist with 27 years’ experience, Polyclinic C5

The participants reported that with every change in medication brand, they would have to spend more time devising methods to communicate the brand change to patients clearly and took longer for patient counselling as well. Given the already existing time constraint, frequent medication brand changes proved to be very challenging. One pharmacist remarked that they had to perform the following additional tasks with every medication brand change:

“So in our clinic, actually we print out a list of new drugs with their drug images and we show the patient, ‘this is the new drug you’re going to take, and the old one looks like this, the new drug looks like this, they’re different.’ If the patient does not understand, we ask them to take a picture, then go back, tell your children that these are different and so on. In addition, then occasionally, we will write for them: ‘This is new.’ And for medications that are new and we know patients will get confused very easily, we will put a label, we will say: ‘this is a change in packaging’ on the label so that whoever is handling that medication for the patients actually know that this medication is a new packaging.”

- Pharmacist with 35 years’ experience, Polyclinic C5

As put across by another pharmacist,

“Oh, we will definitely have to spend a longer time, but there’s no other way.”

- Pharmacist with 27 years’ experience, Polyclinic C5

Even after all these additional efforts put in by the pharmacists, patients would still be confused by medication brand changes. This negated the pharmacists’ previous efforts, affected patient nonadherence, and further increased the workload for the pharmacists and physicians who had to re-explain the brand changes.

“However, sometimes even though we do the counselling and put the ‘change in brand’ sticker, patients are still a bit dubious if they forget about the verbal counselling part. Upon going home, they say: ‘What is this sticker for?’ If they are illiterate, they do not understand the languages that is pasted on the sticker itself, so they will actually just leave the whole pack of medications there and they don’t take them. They will wait until the next visit when they come back and show it to the physician, ‘oh this one I did not take because I do not know what it is for.’”

- Pharmacist with 15 years’ experience, Polyclinic C2

#### Use of video consultation

Video consultations are becoming increasingly popular in Singapore, but pharmacists also raised unique barriers that emerged from them. It was noted that many patients struggled with the usage of technology required for video consultations and that communication over a virtual means was more challenging than face-to-face consultations.

“We have to be a bit more thorough to ensure that they actually capture the information, especially if there’re changes to the medicines itself. So we may take more time for video consult compared to the usual face-to-face consult.”

- Pharmacist with 2 years’ experience, Polyclinic C2 

“They (patients) are not so IT-savvy and they prefer not to use the V-con (video consultation) because they do not know how to use it. In addition, they do not want to trouble their caregivers, or their family members to help them with the setting up and things like that.”

- Pharmacist with 15 years’ experience, Polyclinic C2

### Between pharmacists and physicians

The pharmacists interacted with physicians regularly when managing patients with hypertension; this occurred in both directions, either from physicians to pharmacists or from pharmacists to physicians.

#### From physicians to pharmacists

When physicians made prescriptions or referred patients to pharmacists for medication counselling or reconciliation, they communicated this information to the pharmacists via documentation on electronic medical records (EMRs). Barriers arose from this mode of communication in two ways: lack of EMR integration across institutions and inadequate and inappropriate documentation.

##### Lack of access to patient medication information across institutions

Currently, in Singapore, different public healthcare clusters utilise different EMR platforms. The pharmacists highlighted that they encountered difficulty trying to harmonise a patient’s medications across different healthcare institutions due to the lack of integration of EMRs. When looking for existing or new prescriptions from physicians in other healthcare clusters, pharmacists had to access NEHR (National Electronic Health Record Singapore), which is a completely different portal from SingHealth’s in-house EMR platform.

“SCM is Singhealth cluster’s EMR platform. Then, when you want to find information from other health clusters, you cannot get the information. I mean when patient tell you ‘I recently visited Tan Tock Seng Hospital (TTSH), Khoo Teck Puat Hospital (KTPH)’ OK, you look into NEHR, you see maybe TTSH’s records there, NHG’s (Healthcare group under which TTSH and KTPH fall) records; useful, but still not fully encompassing all the records in Singapore. KTPH I think it uses a different system again that may not even file their medication list.”

- Pharmacist with 10 years’ experience, Polyclinic C3

##### Inadequate and inappropriate documentation by physicians

After making changes or cancellations of medications, some physicians did not document these changes clearly on the EMR. This made it challenging for pharmacists, as they needed to verify the new list of medications or dosages by calling the physicians; this delayed the process of medication review and dispensing.

“Because some of the hospitals’ discharge summaries, especially for those patients who were recently hospitalised, they do not tend to indicate the medication changes. Therefore, it is difficult for us to verify whether the medicine should be continued or discontinued. So if there’s no proper indications, it is a bit hard for us to decide. So we may take more time to actually go through the records.”

- Pharmacist with 2 years’ experience, Polyclinic C2

Beyond delaying medication review and dispensing, this inadequate documentation also led to conflicting advice given to the patient by the pharmacist and the physician.

“Because sometimes there might be a lapse, like for example, the physician told the patient this thing, then after when we counsel the medication to them then they will say but just now the physician said otherwise. However, the physician did not document this information in the clinical documents. Therefore, when I checked as a pharmacist, there was no mention anything like to stop for one day, for example.”

- Pharmacist with 2 years’ experience, Polyclinic C1

In addition to inadequate documentation, the physicians sometimes made mistakes in their documentation on the EMR; this added to the pharmacists’ workload and impacted their roles in medication counselling and dispensing. For instance, some physicians inappropriately documented that their patients needed medication counselling due to their practice of copying and pasting from previous medication instructions in the EMR. This resulted in pharmacists having to routinely reassess their referred patients for the need for medication counselling.

“We will not conduct medication counselling to all the patients who are referred by physicians - we will assess. If let’s say there is no change, no new issue, then we will still send back to our PT (pharmacy technician) to dispense. (These inappropriate documentations arise as) Sometimes they (the physicians) forget, they just copy from the previous (entry). So the previous instructions, they forget to delete.”

- Pharmacist with 12 years’ experience, Polyclinic C1

#### From pharmacists to physicians

##### Phone calls are disruptive and ineffective

In the polyclinic, the pharmacist carries out an ‘intervention’ when they identify an error in the physician’s prescription. The pharmacists would contact and clarify with the physicians before making the appropriate changes; this is often done via a telephone call. The participants expressed that the phone calls frequently disrupted the physician’s consultation with a patient; this resulted in the physician’s delay in picking up the call or acting on the prescription error. With such delays, patients experienced longer waiting times and developed greater dissatisfaction. From the patient’s perspective, the pharmacy staff kept them from leaving the clinic. Therefore, pharmacy staff received the brunt of patient complaints instead of physicians.

“Because sometimes the physicians are seeing patients when the pharmacist calls for intervention. So it may take some time for us to get through to the physicians.”

- Pharmacist with 2 years’ experience, Polyclinic C2

“Because we are the end point, patients will usually tend to blame us (for the delay). Therefore, our colleagues will feel down, and it may affect our pharmacy staff morale.”

- Pharmacist with 2 years’ experience, Polyclinic C1

“Sometimes it can be stressful if the patient is in a rush. They’re pressing for an answer or they might even say, ‘You do not have to check, you just give me the medicine. I know what to do.’ And it is definitely not safe.”

- Pharmacist with 5 years’ experience, Polyclinic C4

Continual disruptions to physicians’ consultations affected the relationship between pharmacists and physicians. This was perceived to be a pertinent problem in a setting where teamwork is essential for a multidisciplinary team to treat patients with hypertension.

“Even though how busy you (physicians) are, try to understand that we (pharmacists) actually do not call you (physicians) for the sake of calling. We do not want to disturb anybody, actually we don’t want to call physicians, we hate to call physicians. [laughter] However, because we have a reason to call you, so maybe just be nicer, try to understand us, why we actually call you.”

- Pharmacist with 27 years’ experience, Polyclinic C5

### Between physicians and patients

The pharmacists highlighted that they were also impacted by the barriers that emerged from communication between physicians and patients. These manifested in a ‘spill-over’ effect, where issues arising from communication between physicians and patients ultimately affected the communication between pharmacists and patients.

#### Language barrier

Pharmacists had to conduct more patient counselling if language barriers during physician‒patient consultations left gaps in the patient’s knowledge. While some pharmacists saw this as part of their role in the multidisciplinary team, others viewed it as an additional burden that added to their already heavy workload. As one pharmacist pointed out,

“Like a Chinese patient sees an Indian physician, for example. There is a language barrier; they don’t understand, they couldn’t request for a Chinese physician and so they may not understand what the physician is trying to say. In the end, they come to the pharmacy, and they found out they are getting one additional medicine. They will ask ‘why am I taking a new medicine?’”

-Pharmacist with 2 years’ experience, Polyclinic C1

#### Patients did not discuss medication nonadherence with physicians

Similarly, when physicians were unable to pick up medication nonadherence, there was an impact on the pharmacist’s workload and decision making. On top of their existing workload, pharmacists had to elicit the reasons for nonadherence, revert to the physician for discussion regarding management options, and advise patients on the importance of adherence.

“When the patient actually tells us that oh, actually, these medications I am not taking at all. Then, I say, you did not tell the physician in the consult room? No, I did not tell the physician. Then, we need to communicate to the physicians. Then, we have to reinforce again that no you cannot self-titrate or self-adjust your dose.”

-Pharmacist with 15 years’ experience, Polyclinic C2

“I’m not sure what happened there in the consultation room. Sometimes they are not so forthcoming. Yeah, so when they come down to the pharmacy, then they tell us there are other problems which they did not tell the physician. These medications could be expensive, he cannot afford or he thinks that his conditions are controlled. So we will document on the thing. We will also discuss this with the physician.”

- Pharmacist with 35 years’ experience, Polyclinic C5

#### Conflicting advice given by physicians

When pharmacists communicated with patients, another barrier emerged when conflicting advice had been given to the patient by the physician. This led to patients becoming nonreceptive to pharmacists’ counselling or even distrusting them.

“Sometimes it’s just a very simple thing like whether this medication can be taken in the morning or night. Just a very simple question. Maybe the physicians did mention it’s better to take at night. Then when they come to me, maybe due to the compliance issue, I would suggest that this medication actually can be taken in the morning together with as your other medication because they tend to forget to take it at night. So for them, they will like, ‘No, the physician says must take at night’. So yes there is some argument there.”

- Pharmacist with 12 years’ experience, Polyclinic C1

On top of this, many pharmacists expressed that patients trusted physicians more than pharmacists. This compounded the effect of conflicting advice, with patients becoming shut-off to pharmacists instead of considering the advice given.

“It is not really whether we are saying the right thing, we’re not saying on the same page and patients feel the difference. Patients think ‘Wow you’re telling me one thing, the physician is telling me another thing, you being the pharmacists, I think you’re not good enough to tell me what I should do.’”

- Pharmacist with 35 years’ experience, Polyclinic C5

## Discussion

This study uncovered the barriers primary care pharmacists in Singapore face in the management of patients with hypertension, with a focus on the challenges arising from communication and documentation. The findings highlighted that pharmacists face difficulties in communicating with both patients and physicians and that physician‒patient communication also has an effect on pharmacists’ management. Team-based care is a promising advancement that taps into the expertise of various healthcare professionals to better control patients’ hypertension [22]. However, working in a team requires good communication between all parties, and this study highlighted several gaps in communication that need to be addressed.

This study identified language as a pertinent barrier that hindered effective communication with and management of patients with hypertension faced by pharmacists. With less than half of Singapore’s population (48.3%) using English as their most frequently spoken language, it is expected that healthcare professionals will meet patients with whom they do not share a spoken language. A study performed among patients in Malaysia, a neighbouring country that also has a multiracial population, reported that nearly all participants felt that the way to improve patient‒physician communication was for physicians to have the capability to speak the local languages [23]. The impact of language barriers has been well documented and includes reducing both patients’ and healthcare professionals’ satisfaction, decreasing the quality of healthcare provided and patient safety, and increasing waiting times for patients while affecting the workflow for healthcare professionals [24–26]. When a healthcare professional and patient lack a common language, initiating the process of shared decision-making, exchanging accurate information, and presenting treatment options can become exceedingly challenging [27].

This study also surfaced the impact of medication brand changes on pharmacists’ management of patients with hypertension. Participants mentioned that due to global supply chain disruptions during the COVID-19 pandemic, the procurement of medications proved to be more challenging, and frequent medication brand changes resulted. However, even after the COVID-19 pandemic, many participants felt that medication brand changes were still a regular occurrence, with some participants pointing to fair trade policies as a cause. Existing interventions for each medication brand change were reported as too time-consuming and ineffective, with many patients still being confused about the changes. There have been few studies on this topic; one study conducted in Sweden [28] found similar challenges faced by pharmacists in terms of generic drug substitution. The study reported that many pharmacists were concerned that their patients would not understand the medication substitutions, leading to disruption of treatment or double medication. This suggests that the challenges faced regarding medication brand changes are widely held and that interventions should be devised to counter this pertinent problem. The Swedish study further surfaced that elderly individuals, who often face a higher degree of polypharmacy (concurrent use of multiple medications commonly defined as five or more), can be a particularly vulnerable group. As Singapore faces an ageing population and with most patients taking chronic hypertension medications being elderly, more emphasis should be placed on addressing the barriers created by frequent medication brand changes. Furthermore, the participants in the Swedish study expressed doubts regarding the actual long-term cost savings for society that are expected from drug brand substitutions. They raised concerns that any potential savings could be offset by the overall rise in medical expenses caused by decreased adherence to medications and the heightened confusion experienced by patients. This was similarly raised in our study, where many pharmacists expressed that even after spending more time and resources trying to help patients understand the medication brand changes, many patients would still be confused. This resulted in decreased medication adherence and increased workload for pharmacists and physicians, who had to perform extensive medication reconciliation and counselling in subsequent clinic follow-ups.

This study also raised certain barriers in communication between pharmacists and physicians, which posed challenges in managing patients with hypertension by pharmacists. One commonly identified barrier was the difficulty pharmacists faced in reaching physicians. Studies conducted in Canada and the United States also pointed out the struggles pharmacists faced in communicating directly with primary care physicians [16, 29]. Notably, many of the pharmacists there felt that a main barrier to communication was the fact that they were not located in the same building as the physicians. The pharmacists from these studies postulated that working in the same physical location would greatly improve communication between physicians and pharmacists.

In Singapore’s polyclinics, physicians and pharmacists work in the same polyclinic, and yet all the participants in our study agreed that getting through to physicians proved to be challenging. Working in the same clinic has overcome the challenge of dealing with “go-betweens” when trying to contact physicians, such as having to navigate clinic answering systems or leaving a message with clinic nurses or receptionists [29], but certain barriers to communication still remain. Pharmacists still raised the concern that physicians would often not respond to their phone calls, a difficulty that has been highlighted in other studies as well [16, 30]. Participants in our study reported that this difficulty in communication translated into longer waiting times for patients, who often became irritable and confrontational to pharmacy staff. This negatively impacted pharmacy staff morale and hindered their workflow. Furthermore, phone calls from pharmacists to physicians often disrupted physicians’ consultations. Pharmacists felt that this annoyed the physicians, resulting in some physicians being rude to the pharmacists. Naturally, this put a strain on the pharmacist-physician relationship and negatively affected team-based care. As one study in Canada reported, physicians and pharmacists both felt that nurturing a healthy pharmacist-physician relationship is important and that this relationship can further improve pharmacist-physician communication [16]. Another study in United States indicates that pharmacists’ understanding of physicians’ preferred mode of communication could enhance communication between them [31]. Thus, one can observe a negative cycle in our study, where the existing means for communication between pharmacists and physicians led to tension between both parties, which further strained the pharmacist-physician relationship. This study also highlighted that barriers emerged when physicians tried to communicate certain information to pharmacists.

Similar to a study conducted in the United States [29], our study surfaced that the main way physicians relayed information to pharmacists was via electronic medical records (EMR) instead of directly over the phone. This means of communication posed a challenge when physicians made inadequate and inappropriate documentation on the EMR. As pointed out by another study, pharmacists mentioned that their conversations with patients were frequently restricted due to inadequate details regarding patient medical conditions, reasons for prescribed medications, and physicians’ treatment strategies [30]. Should pharmacists need clarification regarding physicians’ documentation, they would have to call the physicians, leading to the many challenges raised above.

Finally, our study also pointed out that challenges faced in communication between physicians and patients posed barriers to pharmacists’ management of patients with hypertension. This was observed in two main ways. The first was that physicians sometimes did not elicit patients’ nonadherence to medications. The phenomenon where patients only reveal their difficulties with medication adherence to pharmacists and not physicians has been highlighted in other studies [30, 32]. A systemic review conducted on communication between patients and healthcare professionals revealed that patients might choose not to disclose nonadherence to their physicians out of fear that the physician might react negatively [32]. When our study participants were asked why patients tend not to reveal their medication nonadherence to physicians, some posited that language barriers may have affected physician‒patient communication. This resulted in a “spill-over effect”, where lapses in communication translated into more work for the pharmacists who already faced time constraints. Some pharmacists in our study felt that this negatively impacted the care they could provide for their patients, while others viewed this as an important part of their role in the multidisciplinary team as a “safety net”.

Another barrier faced would be when physicians provide conflicting advice to patients compared to the advice pharmacists provide to patients. The pharmacists highlighted that the advice they provide was tailored to the patients’ context to best achieve medication adherence. However, many participants reported that patients tend to trust physicians more than pharmacists and would not even consider the advice given by pharmacists. In a review looking at challenges faced by pharmacists, some patients were unaware of pharmacists’ increasing role in healthcare and perceived pharmacists as merely dispensers of medicine rather than healthcare partners who collaborate with physicians [33]. This raises the issue of trust that patients place in different healthcare professionals. A study in the United Arab Emirates also pointed out that patients tend not to be receptive to the input of pharmacists when compared to that of physicians [34]. This limited the role a pharmacist can play in a multidisciplinary team and compromised patient care. More research should be conducted on the underlying beliefs and assumptions the public has of allied healthcare professionals, and interventions should be developed to counter any misconceptions to provide the best multidisciplinary care for all patients.

There are several limitations in this study. Firstly, the study was conducted in public primary care clinics in Singapore where there are in-house pharmacists; the findings may not be transferable to practices in the private sector which may not have an in-house pharmacist and has a smaller pool of doctors. Secondly, the interviews were conducted by a single researcher, who is a primary care physician. The clinical role of the interviewer may bring biases during the conduct of the study. However, the interviewer constantly reflected on his role and took steps to avoid leading questions during the interview. The data analysis was also conducted by researchers who were not involved in patient care.

## Conclusions

This study highlights several barriers to communication that pharmacists face when managing patients with hypertension while working in a multidisciplinary team. Communication between pharmacists and patients can be challenging due to patient factors (language barrier and physical disabilities) and clinic factors (medication brand changes, use of video consultations). Barriers to communication between pharmacists and physicians include the existing means of pharmacist intervention (phone calls) being disruptive and ineffective and the prevalence of inadequate and inappropriate documentation on the EMR by physicians. Finally, barriers arising from physician‒patient communication also impact pharmacists, including when patients do not discuss medication nonadherence with physicians and when conflicting advice is given by physicians. These barriers may have a significant impact on patient safety and healthcare professional satisfaction. Thus, interventions involving pharmacists, physicians, and patients, need to be developed. This study has highlighted the need for interventions to help pharmacists support patients cope with medication brand changes. Also, to facilitate better communication between physicians and pharmacists, a more integrated platform that is less disruptive to the clinical workflow should be used. Finally, the electronic medical record system should include a decision support system to reduce physicians’ prescription and documentation errors; this will reduce the burden of pharmacists in rectifying the errors.

## Data Availability

The datasets generated and analysed during the current study are not publicly available due to participant and patient confidentiality.
